# Establishment of Magnetic Microparticles-Assisted Time-Resolved Fluoroimmunoassay for Determinating Biomarker Models in Human Serum

**DOI:** 10.1371/journal.pone.0130481

**Published:** 2015-06-23

**Authors:** Zhi-Qi Ren, Tian-Cai Liu, Si-Hui Zhuang, Guan-Feng Lin, Jing-Yuan Hou, Ying-Song Wu

**Affiliations:** State Key Laboratory of Organ Failure Research, Institute of Antibody Engineering, School of Biotechnology, Southern Medical University, Guangzhou, Guangdong, P. R. China; CRCL-INSERM, FRANCE

## Abstract

In order to early screen and detect suspected biomarkers from pathogens and the human body itself, tracers or reaction strategies that can act as signal enhancers have been proposed forth at purpose. In this paper, we discussed the applicability of magnetic microparticles-assisted time-resolved fluoroimmunoassay (MMPs-TRFIA) for sensitive determination of potential analytes. Hepatitis B e antigen, antibody to hepatitis B surface antigen and free triiodothyronine were used as biomarker models to explore the reliability of the method. By coupling with bioprobes, MMPs were used as immunoassay carriers to capture target molecules. Under optimal condition, assay performance, including accuracy, precision and specificity, was outstanding and demonstrated satisfactory. To further evaluate the performance of the MMPs-TRFIA in patients, a total of 728 serum samples from hospital were analyzed for three biomarkers in parallel with the proposed method and chemiluminescence immunoassay kit commercially available. Fairly good agreements are obtained between the two methods via data analysis. Not only that but the reliability of MMPs-TRFIA has also been illustrated by three different reaction models. It is confirmed that the novel method modified with MMPs has been established and showed great potential applications in both biological detection and clinical diagnosis, including big molecule protein and low molecular weight haptens.

## Introduction

Measurement of different types of serum biomarkers is the foundation of the disease diagnosis and body health. The analysis of biomarkers in human serum offers invaluable information and is essential for the diagnosis of tumor, endocrine, genetic and infectious diseases. Different strategies for rapid and sensitive diagnosis have been discussed and studied increasingly [1–4]. An optimal platform for serology diagnostics enables detection of a broad array of chemical and biomolecules.

At present, most detection technologies developed and employed for immunodiagnostics in hospitals both at home and in abroad are labeling immunoassay technology. This technique, characterized by its high sensitivity and specificity, has been widely employed in basic medicine research and clinical examinations. Recently, enzymatic immunoassay [5–7], chemiluminescence immunoassay (CLIA) [8–10] and time-resolved fluoroimmunoassay (TRFIA) [11–13] have been extensively adopted and acquired widespread applications. However, all these traditional platforms have their inherent limitations. Besides restricted by unsatisfactory sensitivity and linear range [14–16], they are also hard to simultaneously satisfy the requirements of fast, multitarget and high throughput analysis in the latest development. To efficiently tackle these challenges and detect various potential analytes in complex serum samples, here we presented a novel TRFIA modified with magnetic microparticles (MMPs) widely utilized in screening and purification of target analytes.

Traditional TRFIA as a basic platform has been widely used in bioresearch and medicine and rehearsed extensively elsewhere. The highly fluorescent lanthanide (Europium, Eu^3+^) complex, as a non-radioactive tracer, has widely separated excitation and emission wavelengths and long fluorescence life-time [17, 18]. The main benefits of using Eu^3+^ as fluorescent tag include high quantum yield, exceptionally large Stokes shift, narrow emission peaks, optimal excitation and emission wavelengths and minimal background fluorescence for traces detection of chemicals and bacteria [19]. These properties permit measurement of a signal at a time after nonspecific fluorescence has diminished, increasing S/N ratio and enhancing sensitivity [20]. Despite these advantages described in the published literature, undeniably, weaknesses exist in traditional system due to usage of 96-well microtiter plates. The first hurdle is the inefficiency of physical adsorption in coating protein to the surface and that leads to a significant wastage of materials. Furthermore, the amount of analytes that can be detected is insufficient owing to the limited surface area of the wells. In addition, small contact area of the 2D surface of 96-wells results in a time consuming immunoreaction and it generally takes more time to attain equilibrium for an immunoreaction.

To solve these inherent problems, MMPs designed for covalent immobilization of ligands containing–NH_2_ groups were adopted for bioseparation and purification. This chemistry enable ligands to keep their function high and achieve a high yield and low non-specific binding bioseparation. Uniform particle size and superparamagnetic property of the MMPs help good magnetic separation and re-suspension response. These characters of beads are ideal for variety of applications such as isolation and purification of biomolecules from a mixture or analysis of biomolecules. All unique properties of MMPs have gained increasing attention in recent years as a novel functional material. Applications of MMPs in biotechnology and the biomedical sciences have been developed for nearly half a century [21–26]. Compared with conventional liquid-solid phase assays, MMPs demonstrated its superiority in their ability to bind to proteins, promoting reaction speed and sensitivity of searching, detecting and capturing targets. Moreover, more proteins can be bonded to the MMPs by its greater specific surface area of 3D spheroid, contributing to capture more analytes from serum. Magnetic microparticles suspended in the solution with a liquid-liquid phase system provide a large contact area to increase reaction rate and greatly reduce reaction time. In this study, we utilized MMPs as an effective technology tool to detect low levels of antigen-specific antibody with minute sample volumes.

Different categories of analytes, including biomacromolecules and low molecular weight haptens, need different patterns of response to satisfy the constantly improving detection sensitivity. In comparison with biomacromolecules, such as large antigen and antibody (Ag/Ab), haptens are harder to detect due to its few antigenicity domains. Non-competitive immunoassays are the choice of assays for detecting most large antigens owing to its high sensitivity and specificity, simple assay optimization and wide linear range. Competitive immunoassays are still widely used to measure various potential analytes, especially the haptens with a small molecular size [27, 28].

Several research investigated the latest advances in the area of MMPs [29–31]. However, few utilize magnetic particles in TRFIA which applied to the analysis of these biomarkers mentioned in our manuscript. In addition, to date there have been no studies related to the systematic detection of antibodies and low molecular weight haptens by this system. Based on the platform of MMPs-TRFIA, we fabricated large amounts of bioprobes-coated MMPs for the sensitive detection of hepatitis B e antigen (HBeAg) [32], antibody to hepatitis B surface antigen (anti-HBs) [33] and free triiodothyronine (FT4) [34], which were regarded as biomarker model of antigen, antibody and hapten, respectively. Parallel to the proposed method, CLIA for the same analytes in human seurm were performed. By patterning Ag/Ab-coated MMPs and Eu^3+^ chelate-labeled Ag/Ab, we successfully demonstrated that the novel carrier-modified TRFIA could be promisingly applied in the analysis of potential biomolecules (proteins and small metabolites) for biological detection and clinical diagnostics.

## Experimental Section

### Ethics Statement

This study was approved by the Institutional Review Board (IRB) of Nanfang Hospital, Guangzhou, China. A total of 728 human serum samples (257 samples for HBeAg test, 269 samples for anti-HBs test, 202 samples for FT4 test) were kindly donated by patients in Nanfang Hospital.

### Reagents and apparatus

Bovine serum albumin (BSA), 4-morpholineethanesulfonic acid (MES), proclin-300, Tween-20, N-hydroxysulfosuccinimide (NHS), and 1-ethyl-3-(3-dimethylaminopropyl) carbodiimide hydrochloride (EDC) were acquired from Sigma-Aldrich (St. Louis, MO, USA). DTTA-Eu^3+^ (N1-[p-isothiocyannatobenzyl]-diethylene-triamine-N1, N2, N3-tetraacetate-Eu^3+^) (code 1244–302) was obtained from PerkinElmer Life and Analytical Sciences (PerkinElmer, Waltham, MA, USA). Sephadex G-50 was purchased from Amersham Pharmacia Biotech (Piscataway, NJ, USA). MMPs (1101GA-03) were obtained from JSR Life Sciences (Tokyo, Japan). CLIA test kit for three protein markers was obtained from Abbott Laboratories (Chicago, IL, USA). The enhancement solution for Eu^3+^ dissociation and a 1420 Multilabel Counter (Victor^3^TM) were purchased from PerkinElmer Wallac (Turku, Finland). Monoclonal anti-HBeAg antibody (ab11442 and ab113641) produced in mouse were purchased from Abcam (Cambridge, MA, USA). Recombinant HBeAg (ab91273) produced in E. coli was purchased from Abcam (Cambridge, MA, USA). Monoclonal anti-HBs antibody produced in mouse (SAB4700767) was purchased from Sigma-Aldrich (Shanghai, China). Recombinant HBsAg (ab73749) produced in saccharomyces cerevisiae was purchased from Abcam (Cambridge, MA, USA). FT4 (purity, 97%; code 699594) was purchased from Sigma-Aldrich (Shanghai, China). Polyclonal anti-FT4 antibody (ab30833) produced in rabbit was purchased from Abcam (Cambridge, MA, USA). Monoclonal anti-rabbit IgG Fc antibody (RMG02) produced in goat was purchased from Abcam (Cambridge, MA, USA). All other solvents were of analytical grade.

The coating buffer for protein covalently crosslinked to MMPs contained 0.1 mol/L MES (pH 5.0). The blocking buffer was 50 mmol/L Tris–HCl, 0.9% NaCl, 0.04% NaN_3_, 2% BSA (pH 8.0). Washing buffer was 0.05 mol/L Tris–HCl, 0.01% Tween-20, and 0.15 mol/L NaCl (pH 7.8). Tris-buffered saline Tween-20 (TBST) was the solution for preservation of immunomagnetic microparticles, containing 0.025 mol/L Tris, 0.15 mol/L NaCl and 0.05% Tween-20 (pH 7.4). Eu^3+^ chelate-labeling buffer was 0.05 mol/L Na_2_CO_3_ (pH 9.0). TRFIA assay buffer used to dilute the Eu^3+^ chelate-labeled Ag/Ab comprised 0.05 mol/L Tris–HCl buffer, 1.5% polyethyleneglycol 6000, 0.3 mmol/L BSA, 0.01% Proclin-300, 0.15 mol/L NaCl, 0.02% (w/v) bovine globulin and 0.01% Tween-20 (pH 7.8).

### Synthesis of protein-conjugated magnetic microparticles

Immunomagnetic microparticles conjugates were synthesized by a standard protocol recommended by the manufacturer. Firstly, carboxyl groups on the surface of MMPs must be activated in a way. Our processing flow included adding 25 μl fresh EDC (10 mg/ml) and 40 μl NHS (10 mg/ml) to 10 mg (100 mg/ml, 10.0×10^9^ magnetic particles/ml in H_2_O) of carboxyl-modified MMPs in 1 ml coating buffer, and rotating (600 rpm) end-over-end for 30 min at 25°C. Then MMPs were removed from the magnetic field and washed thrice to remove excess activator. After that, appropriate amount (100 μg) of purified Ag/Ab in 1 ml binding buffer was added to the activated MMPs and mixed by gentle rotation (600 rpm) overnight at 25°C. Once MMPs-Ag/Ab conjugates were synthesized, they were blocked with 1 ml blocking buffer for 3 h, then washed thrice with TBST and stored in the same solution at 4°C until use.

### Labeling of Eu^3+^ chelate-labeled Ag/Ab

Follow a similar approach for the synthesis of Eu^3+^ chelate-labeled Ag/Ab. First, 1 mg of purified Ag/Ab in chelate-labeling buffer was added with 0.2 mg of DTTA-Eu^3+^. After being fully mixed, the mixture was incubated and should be kept away from light for 24 h at room temperature. Then free Eu^3+^-chelates were separated from the tracer by size exclusion chromatography on a Sephadex G-50 column (1.5 cm × 40 cm). After eluting with spent regenerant, about six milliliters of tracer solution was gathered. To stabilize the tracer solution, we added BSA to give a final concentration of l mg/ml and stored the preparation at 4°C and were stable for 12 months by adding 0.2% BSA as stabilizer.

### Statistics

For each assay, samples were tested in triplicate and experiments were repeated at least three times. By using buffer instead of the primary antibody, background measurements were derived from each experiment. Its value was calculated as the mean fluorescence intensity of three wells minus background ± standard deviation. For comparison, we adopted commercial CLIA kit that performed exactly according to the instructions obtained by the manufacturers. McNemar’s test and Pearson's correlation were performed to show the linearity and correlations between two methods. Calibration curves were obtained by plotting the logarithm of fluorescence intensity (Y) against the logarithm of analyte concentration (X) and fitting a logistic equation using Origin Pro7.5 (Microcal, USA): Log (Y) = A + B × Log(X). Statistical analysis performed using McNemar’s test by SPSS 13.0 (Chicago, IL, USA), Microsoft Excel 15.0 (Microsoft, Redland, WA).

### MMPs-TRFIA measurement

**Fig 1:**
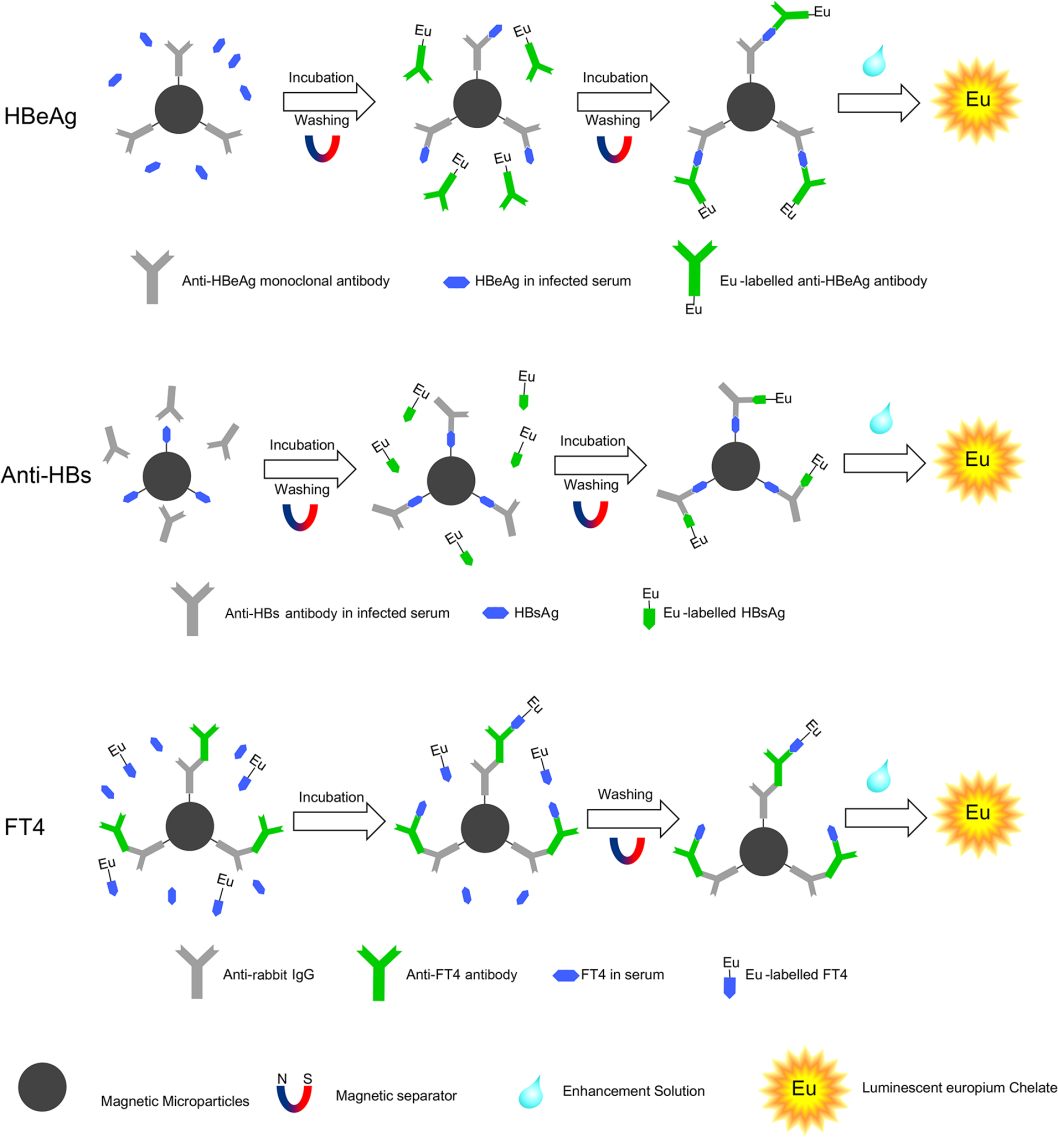
Schematic diagram of the MMPs-TRFIA for determinations of HBeAg, Anti-HBs and FT4.

The novel protocol was carried out by combining different strengths of MMPs and lanthanide chelates. HBeAg, anti-HBs and FT4 were determinated by double antibody sandwich method, double antigen sandwich method and competitive inhibition method, respectively (Fig 1). These assays were all done in triplicate. Serial standards of HBeAg and anti-HBs were diluted in normal human plasma and standards of FT4 were diluted in non-hormone serum. HBeAg and anti-HBs quantitative assays were both performed as a two-step procedure and they carried out through the following the steps. In the first step, 100 μl serum samples or standards and 50 μl of appropriate dilution of magnetic microparticles were added to 96-well plate. Plates were incubated while being agitated 600 rpm for 15 min. After incubation, place the plate on the magnetic separator and remove the supernatant carefully. After washing four times, 100 μl of europium labelled Ag/Ab were added to the plates followed by incubation for another 15 min at 600 rpm. After washing six time again, enhancement solution, which dissociates europium ions from the bound labelled Ag/Ab, was added to suspend the beads and then formed highly fluorescent chelates. HBeAg and anti-HBs were quantified based on the fluorescence measured using the Victor3 1420 Multilabel Counter at an excitation wavelength of 340 nm and emission wavelength of 613 nm. The delay time was 400μs. In another part of our research, the FT4 was tested by one-step heterologous competitive method between FT4 in sample (25μl) and Eu-labelled anti-T4 antibody (100μl). They were incubated simultaneously at 25°C for 30 min. Similarly, after washing six times, Europium was dissociated by adding enhancement solution to each well. The fluorescence measurement and data process were done in the same way as described above. Their concentrations were automatically calculated from the respective index calibration curve. The calibration curves were obtained by plotting the fluorescence readings of standards against the respective concentration using Origin 7.5.

### Analytical method for performance evaluation

Calibration curves for HBeAg, anti-HBs and FT4 were created by measuring a series of known concentrations of standards. Calibration curves of HBeAg and anti-HBs were graphed by plotting the logarithm of fluorescence intensity (Y) against the logarithm of analyte concentration (X) and calibration curve of FT4 was obtained by plotting logit-log against the decimal log values of the corresponding standard concentrations. B_0_ and B are defined as the fluorescence signals from tracer bound to MMPs at the zero FT4 standard and at the other FT4 standards, respectively. Logit-log plot is obtained from the computation formula: logit = ln[(B/B_0_)/(1-B/B_0_)]. The curves and logistic equations were obtained using Origin Pro7.5 (Microcal, USA): Log (Y) = A + B × Log(X). The functional detection limits, defined as the lowest analyte concentration that could be measured with a CV of 15% or less in ten replica measurements. The accuracy was calculated by measuring analytes at the conditions of high, middling and low of substrate concentrate and the results were compared to the quantity's actual value. The precision of a measurement system, related to reproducibility and repeatability, is the degree to which repeated measurements under unchanged conditions show the same results. The precision depends on the repeatability of quantitative results obtained by three serum samples and the same batch of reagents over different time periods. The specificity or anti-interference-ability was quantified by measuring a range of similar analytes or possible interfering compounds relative to the target analytes. Finally by compared with commercial CLIA kit, a large number of serum samples were tested. McNemar’s test and Pearson's correlation were performed to show the linearity and correlations between two methods. The application of the innovative method in clinical diagnosis, prognosis and therapeutic monitoring and blood screening was discussed from two aspects of theoretics and experimentation.

## Results and Discussions

### Labeling rate of protein-coated MMPs

The determination method of protein content on the surface of MMPs by bicinchoninic acid (BCA) method was set up. By the determination of purified protein-coated MMPs, about 7.8 μg protein was coupled to per mg MMPs, namely the labeling yield was about 78% and the immunological reaction activity was kept at high level.

### Optimization of the assays

Various reaction parameters, including incubation time, the concentration of magnetic and dilution ratio of Eu3+-labeled antibody or antigen, were studied to assess its influence on assay performance. The optimum conditions were obtained through the orthogonal experiment. With the optimization of conditions, the results indicated that the fluorescence intensity increased with incubation time but did not reach a dynamic balance until 30 min and were depicted in S1 Fig. Finally, within the time range considered, 30 min was selected as the optimum reaction time for analysis of three biomarkers in subsequent work. In addition, a higher fluorescence intensity was achieved with an increase in the amount of MMPs and Eu^3+^-labeled proteins. Applying the orthogonal analytic method, when concentration of MMPs and dilution ratio of Eu^3+^-labeled anti-HBe antibody reached 400 μg/mL and 1/25, respectively, the value was no longer increasing significantly and details were provided in S1 Table. Thus, 400 μg/mL of MMPs and a dilution ratio of 1/25 were selected as the optimal condition for HBeAg assay. Similarly, 300 μg/mL of MMPs and a dilution ratio of 1/50 were selected as the optimal condition for anti-HBs assay and details were given in S2 Table. Then, 300 μg/mL of MMPs and a dilution ratio of 1/25 was selected for FT4 assay and details were given in S3 Table. Through studying and optimizing process, performance of the MMPs-TRFIA was evaluated through the optimum reaction conditions in subsequent work.

### Performance of the MMPs-TRFIA

**Fig 2:**
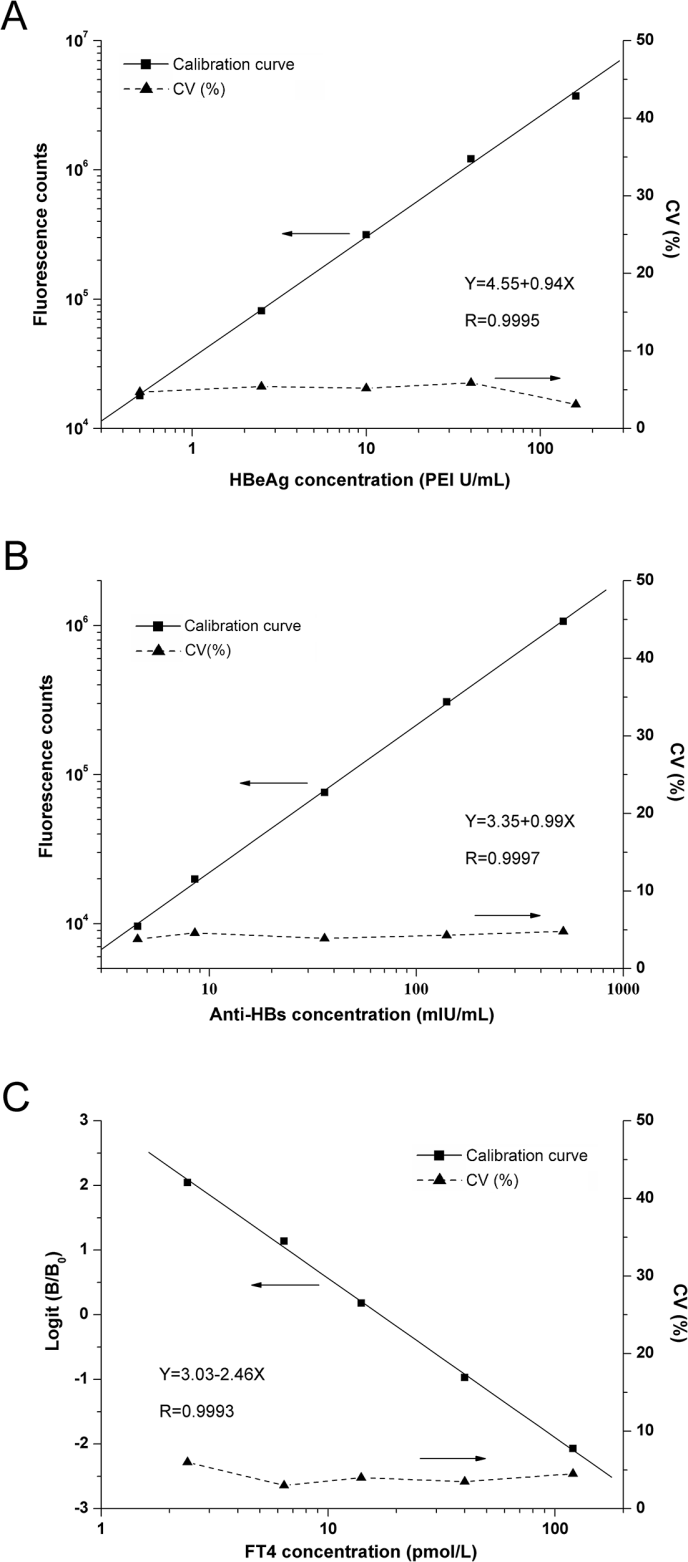
Calibration curves for MMPs-TRFIA for determinations of HBeAg (A), Anti-HBs (B) and FT4 (C) standards. Fluorescence intensity and standard deviations were calculated from a set of five measurements.

**Fig 3:**
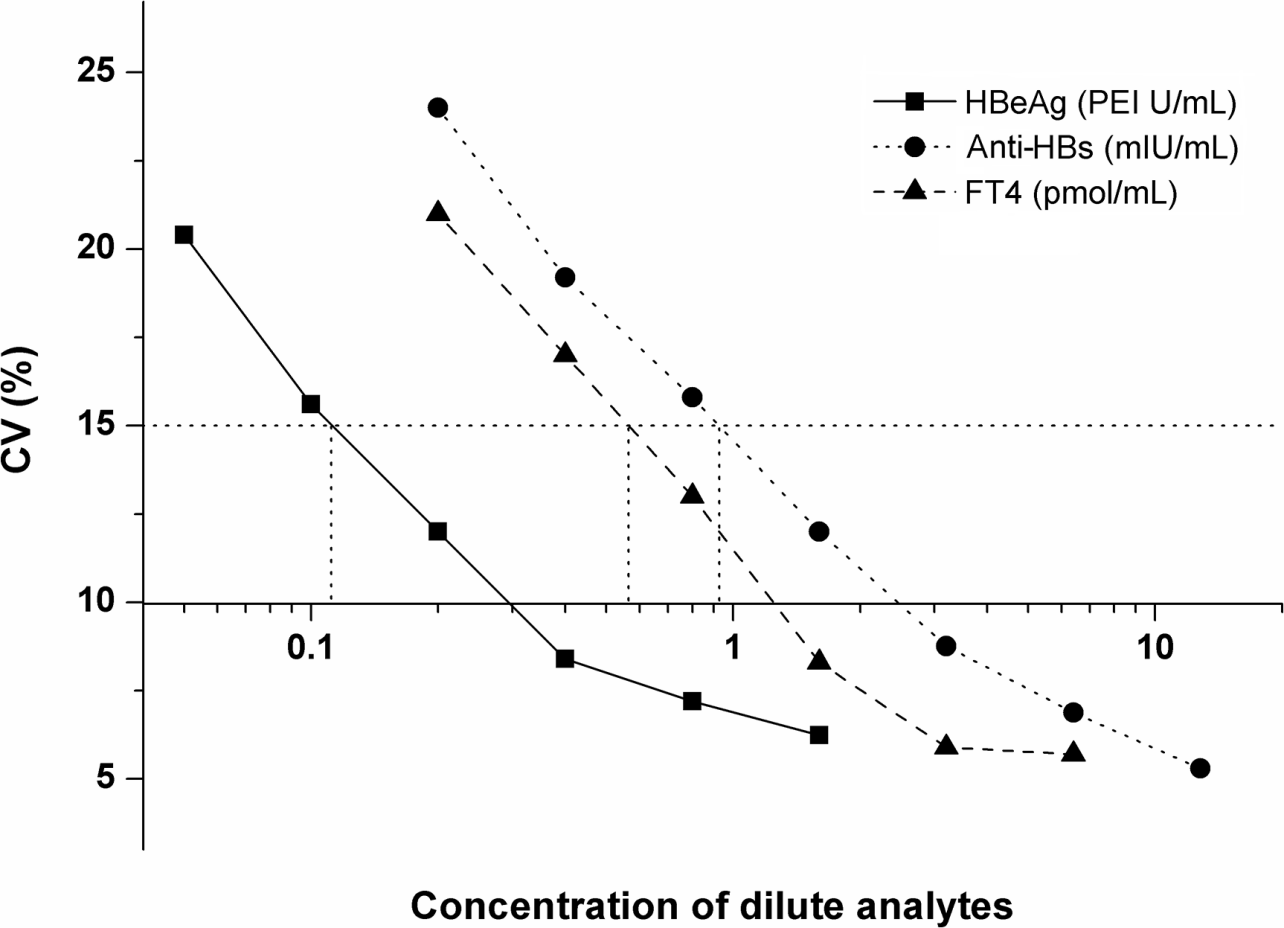
Functional detection limits of HBeAg, Anti-HBs and FT4 were the lowest analyte concentration that could be measured with a CV of 15% or less in ten replica measurements.

Under optimal condition, the calibration curves for HBeAg (0, 0.5, 2.5, 10, 40, 160 PEI U/mL), anti-HBs (0, 4.5, 8.5, 36, 140, 513 mIU/mL) and FT4 (0, 2.4, 6.4, 14, 40 and 120 pmol/L) as biomarker models following our protocol were well displayed in Fig 2. Calibration curves of HBeAg and anti-HBs were carried out using log-log regression and the FT4 linearized standard curve was obtained by plotting logit (B /B_0_). The best-fit calibration was determined to be described by the following equation (HBeAg: log(Y) = 0.94 log(X) + 4.55 with a correlation coefficient of 0.9995; anti-HBs: log(Y) = 0.99log(X) + 3.35 with a correlation coefficient of 0.9997; FT4: logit(B/B_0_) = 3.03–2.46log(X) with a correlation coefficient of 0.9993). The calibration curve for HBeAg showed a linear relationship over the concentration range of 0.2–160 PEI U /mL and its functional detection limit was estimated to be approximately 0.12 PEI U /mL for HBeAg, 0.95 mIU/mL for anti-HBs, 0.47 pmol/L for FT4, respectively (Fig 3). Similarly, anti-HBs had a wide linear range of 1.5–600 mIU/mL and a functional detection limit of 0.95 mIU/mL. FT4 had a wide linear range of 1.0–200 pmol/L and a functional detection limit of 0.47 pmol/L. Such low detection limits and wide linear ranges are comparable with or better than other methods previously reported. A comparison with several methods is shown in Table 1.

**Table 1:** Comparison of sensitivity and linear range for the determination of HBeAg, Anti-HBs and FT4 by previously reported methods.

Target analytes	Methods	Detection limit	Linear range
HBeAg (PEI U/mL)	RIA [14]	0.5	0.5–12
	Chemiluminescence ELISA [14]	0.5	0.5–50
	Fluorescence ELISA [14]	0.5	0.5–100
	proposed method	0.12	0.2–160
Anti-HBs (mIU/mL)	CE-CL [15]	1	2–200
	proposed method	0.95	1.5–600
FT4 (pmol/L)	TRFIA [16]	0.6	2.5–120
	MPs-CLEIA [35]	0.25	1.59–122
	MCE-CL [36]	2.2	5–250
	proposed method	0.47	1–200

Abbreviation: RIA, Radioimmunoassay; Chemiluminescence ELISA, chemiluminescence enzyme-linked immuno sorbent assay; Fluorescence ELISA, Fluorescence enzyme-linked immunosorbent assay; CE-CL, capillary electrophoresis with chemiluminescence; MPs-CLEIA, Magnetic particle-based chemiluminescence enzyme immunoassay; MCE-CL, microchip electrophoresis chemiluminescence

### Assay accuracy and precision

Recovery (ε = a/A, a is the observed value, A is the actual value) and coefficient of variation (CV = σ/μ, σ and μ represent the standard deviation and the mean of a range of values) were calculated to evaluate the accuracy and precision of the novel method. 90% ≤ε ≥ 110% and coefficients of variation (CV) ≤10% were considered statistically acceptable. Three levels of HBeAg, anti-HBs and FT4 in the assay buffer were measured to assess the detection accuracy. The precision of the assays was performed by measuring three serum samples in ten consecutive analytical runs in one day and lasting for five separate days. The average recoveries of HBeAg, anti-HBs and FT4 were 98.0–99.7%, 96.4–101.8% and 93.4–97.3%, respectively (Table 2). The intra-assay and inter-assay CV were <4.3% and <7.8%, respectively (Table 3). After serial dilution tests, the measurements showed good agreement between the expected and measured values for three analytes and were all in normal range.

**Table 2:** Accuracy of MMPs-TRFIA was calculated by measuring seril dilutions of known concentration substances and these results were compared to the quantity's actual value.

Samples	Expected	Observed	Recovery (%)
HBeAg (PEI U/mL)	1.5	1.47	98.0
	12.5	12.42	99.4
	100	99.69	99.7
Anti-HBs (mIU/mL)	9.5	9.67	101.8
	37.5	36.15	96.4
	300	296.57	98.9
FT4 (pmol/L)	5	4.67	93.4
	20	19.45	97.3
	100	96.63	96.6

**Table 3:** Assay precision: intra-assay and inter-assay stability of the MMPs-TRFIA.

Concentration of samples		Intra-assay precision^a^ (n = 8)		Inter-assay precision^a^ (n = 8)	
		Measured (IU/mL)	CV(%)^b^	Measured (IU/mL)	CV(%)^b^
HBeAg (PEI U/mL)	1.5	1.40±0.04	2.8	1.49±0.09	6.0
	12.5	12.3±0.53	4.3	12.12±0.60	5.0
	100.0	95.15±3.89	4.1	98.62±4.12	4.2
Anti-HBs (mIU/mL)	9.5	9.01±0.22	2.4	9.02±0.32	3.5
	37.5	37.35±1.59	4.3	36.43±1.81	5.0
	300.0	290.37±10.56	3.6	290.78±11.79	4.1
FT4 (pmol/L)	5.0	4.97±0.16	3.2	5.00±0.29	5.8
	20.0	19.27±0.83	4.3	19.66±1.37	7.0
	100.0	94.37±2.56	2.7	97.42±3.93	4.0

^a^ Mean value ± standard deviation (SD)

^b^CV = (SD/Mean)×100%

Abbreviation: CV, coefficient of variation

### Assay specificity

To estimate the specificity of antibody or antigen, a diverse array of cross-reactants relative to HBeAg, anti-HBs and FT4 were tested for the immunoassay. The cross-reactivity (CR%) was calculated by the formula as follows: cross-reactivity (%) = (determined concentration of analytes)/(actual concentration of cross-reactant). All of cross-reactivity in Table 4 were very low and proved that the cross-reactants show no cross-reaction to these three biomarkers. The response of targets could be discriminated obviously from those cross-reactants. Thus, the assay exhibited high specificity to targets but little cross-reactivity with some compounds structurally similar to them. The selectivity of the novel TRFIA was acceptable and it can be employed in screening immunoassays to detect various biomarkers.

**Table 4:** Assay specificity: effect of potentially interfering substances on the determination of HBeAg, Anti-HBs and FT4.

Target analytes	Cross-reactants	Added values	Determined values	CR (%)
HBeAg	HBsAg	200 IU/mL	0.17 PEI U/mL	0.085
	HBcAg	100 ng/mL	0.15 PEI U/mL	0.15
Anti-HBs	HBeAb	100 PEIU/ml	1.1 mIU/ mL	1.1
	HBcAb	100 PEIU/ml	1.0 mIU/mL	1.0
FT4	T3	40 pmol/L	0.27 pmol/L	0.67
	rT3	40 pmol/L	0.38 pmol/L	0.95

Abbreviation: HBsAg, hepatitis B surface antigen; HBeAb, hepatitis B e antibody; HBcAg, hepatitis B core antigen; HBcAb, hepatitis B core antibody; T3, 3,3’,5-Triiodo-L-thyronine; rT3, 3,3’,5’-Triiodo-L-thyronine.

### Clinical serum samples

**Fig 4:**
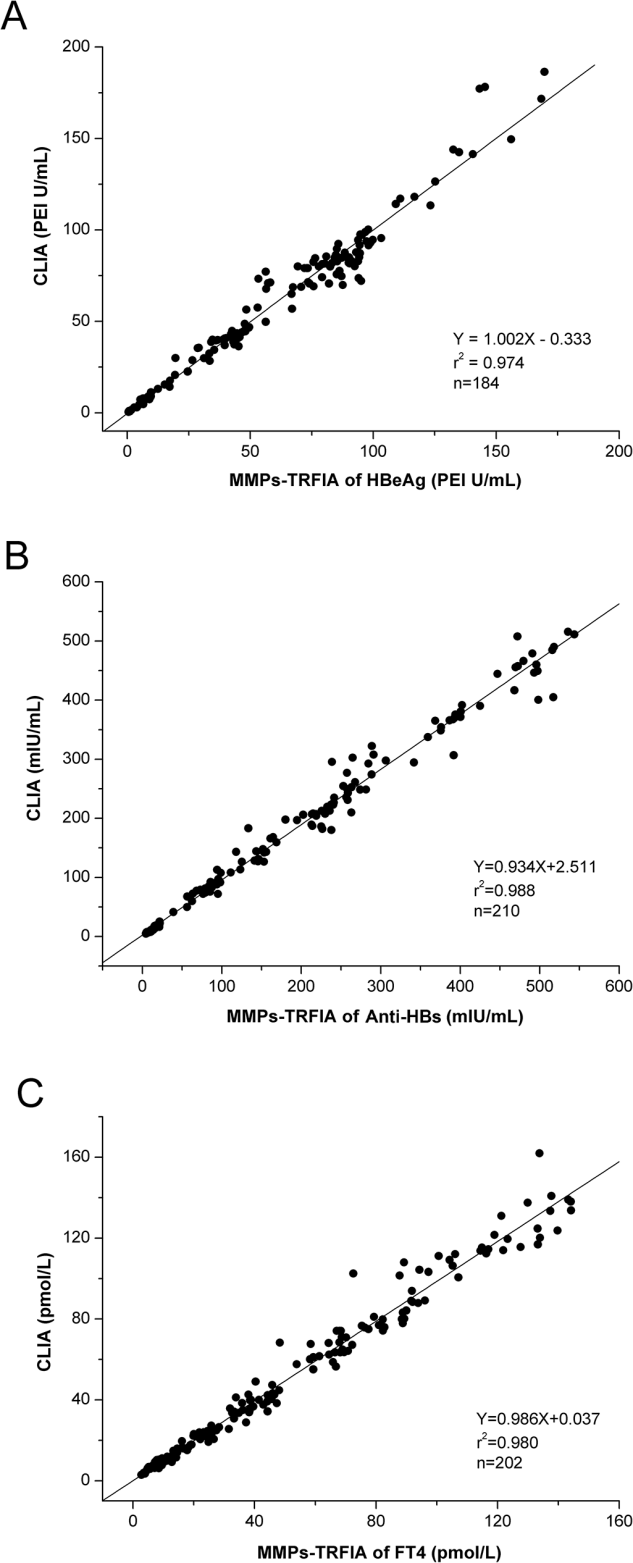
Plot of the results obtained with the proposed method versus those obtained using CLIA for 184 positive HBeAg samples (A), 210 positive anti-HBs samples (B) and 202 FT4 samples(C).

To further demonstrate the reliability of the MMPs-TRFIA for the determination of HBeAg, anti-HBs and FT4, a total of 728 serum samples (257 samples for HBeAg test, 269 samples for anti-HBs test, 202 samples for FT4 test) were all analyzed in parallel with the proposed method and commercially available CLIA kit. Difference in results of HBeAg and anti-HBs testing between the two methods were evaluated with McNemar’s test for correlated proportions (Tables 5 and 6). Sensitivity and specificity between tests were obtained by McNemar’s test. Compared with CLIA, the MMPs-TRFIA of HBeAg had a sensitivity of 98.9% and a specificity of 95.8%, and Kappa value was 0.951. Five discrepant HBeAg samples were rechecked by fluorescence quantitative PCR. Results of three were in agreement with proposed method and the other two were in agreement with CLIA. According to Table 6, the anti-HBs had a sensitivity of 98.1% and a specificity of 96.3%, and Kappa value was 0.932. Six discrepant anti-HBs samples were rechecked by TRFIA. Results of four were in agreement with proposed method and the other two were in agreement with TRFIA. In addition, of all those samples, the positive samples confirmed by two methods (184 positive samples for HBeAg, 210 positive samples for anti-HBs) and all 202 FT4 samples were selected to demonstrate the correlation between two methods using linear regression analysis. The correlations between them were observed as shown in Fig 4. Analytes levels measured by both methods were very close and correlations gave a good agreement between the two assays (HBeAg: Y = 1.002X-0.333, r^2^ = 0.974, P<0.001; anti-HBs: Y = 0.934X+2.511, r^2^ = 0.988, P<0.001; FT4: Y = 0.986X+0.037, r^2^ = 0.980, P<0.001). As shown in Fig 4, there was a linear relativity between the results obtained with the two methods when the assays were performed in duplicate at the same time. Good agreements were achieved in all patient groups. The described method has the advantages of high sensitivity and specificity and provides a useful tool for the detection of biomarkers in human serum. Thus, the proposed method presented here could be a sensitive and efficient diagnosis system for accurate blood screening and disease surveillance.

**Table 5:** McNemar’s test between the proposed method and CLIA for the test of 257 HBeAg samples.

		CLIA		
		+	-	Total
Proposed Method	+	184	3	187
	-	2	68	70
	Total	186	71	257

Positive/Negative: +/-

**Table 6:** McNemar’s test between the proposed method and CLIA for the test of 269 anti-HBs samples.

		CLIA		
		+	-	Total
Proposed Method	+	210	2	212
	-	4	53	57
	Total	214	55	269

Positive/Negative: +/-

## Conclusions

Magnetic microparticles binded with specific antibodies have widely penetrated into microbiology, biochemistry, molecular genetics and other fields, specifically including applications of immunoassay, cell separation and biomacromolecule purification. The microparticles we used are well-designed MMPs for covalent immobilization of ligands containing–NH_2_ groups. We presented a novel sensitive method based on MMPs and TRFIA for the analysis of biomarker models in human serum, including antigen, antibody and low molecular weight haptens. It contributed to a significant improvement in sensitivity compared with the conventional TRFIA and CLIA. The results of our study fulfilled the system of MMPs-TRFIA for determinating three categories of analytes in the presence of higher demands on rapid, accurate and precise detection. With the utilization of MMPs and Eu^3+^ chelate, the MMPs-assisted TRFIA achieved high sensitivity and wider linear range. It is our belief that the novel technology platform combined with high speed and sensitivity seen in this and our previous studies [37] has great potential in rapid and high-throughput immunoassay testing and will bring about major improvements in the in vitro diagnostic and monitoring of patients with a wide range of clinical conditions.

## Supporting Information

S1 Fig Optimization of the incubation time in detection of HBeAg, anti-HBs and FT4. Experimental conditions: The curves correspond to a series of incubation times (from 10 to 60 min), 50 μL of magnetic particles (500 μg/mL), 100 μL of Eu^3+^-labeled antibody (dilution ratio of 1:10). The standard sample of HBeAg (160 PEI U/mL), anti-HBs (513 mIU/mL) and FT4 (120 pmol/L) were used to assess the influence of incubation time to the test. The results indicated that the fluorescence intensity increased with incubation time but did not reach a dynamic balance until 30 min. Finally, within the time range considered, 30 min was selected as the optimum reaction time in subsequent work.
(TIF)

S1 Table Optimization of HBeAg assay: the concentration of dilution MMPs and ratios of Eu^3+^-labeled anti-HBe antibody. The optimal conditions were obtained by orthogonal analyses on the data of the experiment. As shown in S1 Table, a higher fluorescence intensity was achieved with an increase in the amount of MMPs and Eu^3+^-labeled anti-HBe antibody. As we could see, based on data analysis, when concentration of MMPs and dilution ratio of Eu^3+^-labeled anti-HBe antibody reached 400 μg/mL and 1/25, respectively, the value was no longer increasing significantly. Thus, 400 μg/mL of MMPs and a dilution ratio of 1/25 was selected as the optimal condition for HBeAg assay.
(DOC)

S2 Table Optimization of anti-HBs assay: the concentration of dilution MMPs and ratios of Eu^3+^-labeled HBsAg. The optimal conditions were obtained by orthogonal analyses on the data of the experiment. Similarly, as shown in S2 Table, when the concentration of MMPs and dilution ratios of Eu^3+^-labeled HBsAg reached 300 μg/mL and 1/50, respectively, the fluorescence intensity was no longer increasing significantly. Thus, 300 μg/mL of MMPs and a dilution ratio of 1/50 was selected as the optimal condition for anti-HBs assay.
(DOC)

S3 Table Optimization of FT4 assay: the concentration of dilution MMPs and ratios of Eu^3+^-labeled T4. The optimal conditions were obtained by orthogonal analyses on the data of the experiment. Similarly, as shown in S3 Table, when the concentration of MMPs and dilution ratios of antibody reached 300 μg/mL and 1/25, respectively, the fluorescence intensity was no longer increasing significantly. Thus, 300 μg/mL of MMPs and a dilution ratio of 1/25 was selected as the optimal condition for FT4 assay.
(DOC)
