# Supplementary figures and images for: Establishment of Magnetic Microparticles-Assisted Time-Resolved Fluoroimmunoassay for Determinating Biomarker Models in Human Serum
Source: PLoS One. 2015 Jun 23;10(6):e0130481. doi: 10.1371/journal.pone.0130481 (PMC4478010; doi:10.1371/journal.pone.0130481)

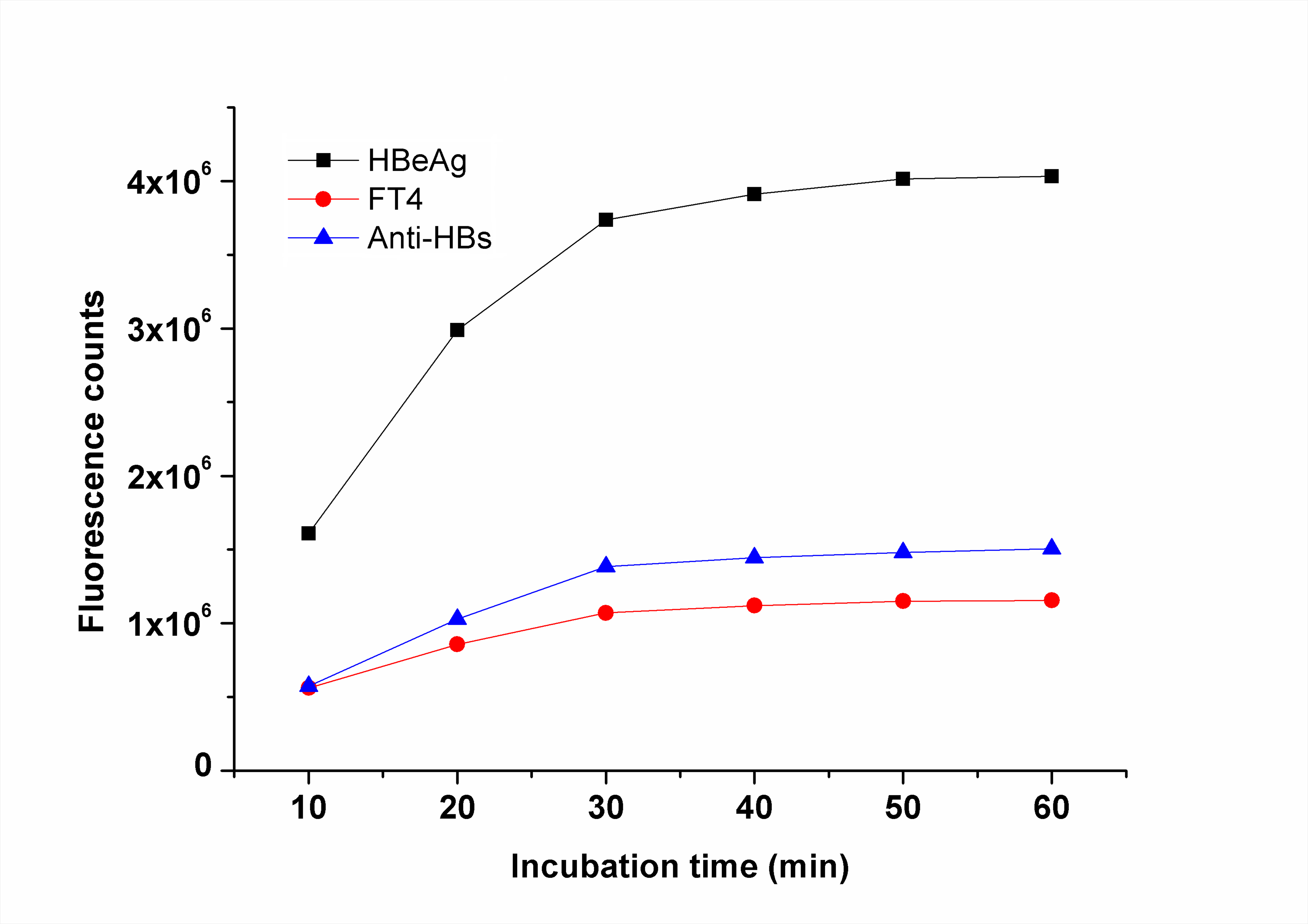

Supplement: S1 Fig — Experimental conditions: The curves correspond to a series of incubation times (from 10 to 60 min), 50 μL of magnetic particles (500 μg/mL), 100 μL of Eu3+-labeled antibody (dilution ratio of 1:10). The standard sample of HBeAg (160 PEI U/mL), anti-HBs (513 mIU/mL) and FT4 (120 pmol/L) were used to assess the influence of incubation time to the test. The results indicated that the fluorescence intensity increased with incubation time but did not reach a dynamic balance until 30 min. Finally, within the time range considered, 30 min was selected as the optimum reaction time in subsequent work. (TIF) [file pone.0130481.s001.tif]
