# Supplementary material for: Establishment of Magnetic Microparticles-Assisted Time-Resolved Fluoroimmunoassay for Determinating Biomarker Models in Human Serum
Source: PLoS One. 2015 Jun 23;10(6):e0130481. doi: 10.1371/journal.pone.0130481 (PMC4478010; doi:10.1371/journal.pone.0130481)
Supplement: S1 Table — The optimal conditions were obtained by orthogonal analyses on the data of the experiment. As shown in S1 Table, a higher fluorescence intensity was achieved with an increase in the amount of MMPs and Eu3+-labeled anti-HBe antibody. As we could see, based on data analysis, when concentration of MMPs and dilution ratio of Eu3+-labeled anti-HBe antibody reached 400 μg/mL and 1/25, respectively, the value was no longer increasing significantly. Thus, 400 μg/mL of MMPs and a dilution ratio of 1/25 was selected as the optimal condition for HBeAg assay. (DOC) [file pone.0130481.s002.doc]

**Table S1. Optimization of HBeAg assay: the concentration of** **dilution MMPs and ratios of** **Eu^3+^-labeled anti-HBe antibody.**

|  | | Dilution ratios of Eu^3+^-labeled anti-HBe antibody | | | | |
| --- | --- | --- | --- | --- | --- | --- |
|  |  | 1/200 | 1/100 | 1/50 | 1/25 | 1/10 |
| Concentration of MMPs (µg/mL) | 100 | 794811 | 1344896 | 1520614 | 1644895 | 1714691 |
|  | 200 | 1264927 | 1669623 | 2059408 | 2452372 | 2642214 |
|  | 300 | 1582854 | 2169115 | 2699485 | 3108563 | 3178349 |
|  | 400 | 1754820 | 2449497 | 3353265 | 3737198 | 3839286 |
|  | 500 | 1895209 | 2652374 | 3506892 | 3823935 | 3925970 |

The optimal conditions were obtained by orthogonal analyses on the data of the experiment. As shown in Table 1, a higher fluorescence intensity was achieved with an increase in the amount of MMPs and Eu^3+^-labeled anti-HBe antibody. As we could see, based on data analysis, when concentration of MMPs and dilution ratio of Eu^3+^-labeled anti-HBe antibody reached 400 µg/mL and 1/25, respectively, the value was no longer increasing significantly. Thus, 400 µg/mL of MMPs and a dilution ratio of 1/25 was selected as the optimal condition for HBeAg assay.
