# Supplementary material for: Establishment of Magnetic Microparticles-Assisted Time-Resolved Fluoroimmunoassay for Determinating Biomarker Models in Human Serum
Source: PLoS One. 2015 Jun 23;10(6):e0130481. doi: 10.1371/journal.pone.0130481 (PMC4478010; doi:10.1371/journal.pone.0130481)
Supplement: S2 Table — The optimal conditions were obtained by orthogonal analyses on the data of the experiment. Similarly, as shown in S2 Table, when the concentration of MMPs and dilution ratios of Eu3+-labeled HBsAg reached 300 μg/mL and 1/50, respectively, the fluorescence intensity was no longer increasing significantly. Thus, 300 μg/mL of MMPs and a dilution ratio of 1/50 was selected as the optimal condition for anti-HBs assay. (DOC) [file pone.0130481.s003.doc]

**Table S2. Optimization of anti-HBs assay: the concentration of dilution MMPs and ratios of Eu^3+^**-labeled HBsAg.

|  | | Dilution ratios of Eu^3+^-labeled HBsAg | | | | |
| --- | --- | --- | --- | --- | --- | --- |
|  |  | 1/200 | 1/100 | 1/50 | 1/25 | 1/10 |
| Concentration of MMPs (µg/mL) | 100 | 357924 | 527958 | 608246 | 627958 | 655876 |
|  | 200 | 515971 | 667849 | 823763 | 980949 | 1056886 |
|  | 300 | 633142 | 867646 | 1109794 | 1143425 | 1171340 |
|  | 400 | 651928 | 919799 | 1141306 | 1194879 | 1215714 |
|  | 500 | 688084 | 936095 | 1192757 | 1209574 | 1223880 |

The optimal conditions were obtained by orthogonal analyses on the data of the experiment. Similarly, as shown in Table 2, when the concentration of MMPs and dilution ratios of Eu^3+^-labeled HBsAg reached 300 µg/mL and 1/50, respectively, the fluorescence intensity was no longer increasing significantly. Thus, 300 µg/mL of MMPs and a dilution ratio of 1/50 was selected as the optimal condition for anti-HBs assay.
