# Supplementary material for: Establishment of Magnetic Microparticles-Assisted Time-Resolved Fluoroimmunoassay for Determinating Biomarker Models in Human Serum
Source: PLoS One. 2015 Jun 23;10(6):e0130481. doi: 10.1371/journal.pone.0130481 (PMC4478010; doi:10.1371/journal.pone.0130481)
Supplement: S3 Table — The optimal conditions were obtained by orthogonal analyses on the data of the experiment. Similarly, as shown in S3 Table, when the concentration of MMPs and dilution ratios of antibody reached 300 μg/mL and 1/25, respectively, the fluorescence intensity was no longer increasing significantly. Thus, 300 μg/mL of MMPs and a dilution ratio of 1/25 was selected as the optimal condition for FT4 assay. (DOC) [file pone.0130481.s004.doc]

**Table S3. Optimization of FT4 assay: the concentration of dilution MMPs and ratios of Eu^3+^**-labeled T4.

|  | | Dilution ratios of Eu^3+^-labeled T4 | | | | |
| --- | --- | --- | --- | --- | --- | --- |
|  |  | 1/200 | 1/100 | 1/50 | 1/25 | 1/10 |
| Concentration of MMPs (µg/mL) | 100 | 364826 | 610400 | 688846 | 744328 | 775487 |
|  | 200 | 574700 | 755367 | 929379 | 1194809 | 1279560 |
|  | 300 | 716631 | 968355 | 1215127 | 1387751 | 1418906 |
|  | 400 | 743402 | 1013525 | 1256993 | 1428392 | 1453967 |
|  | 500 | 766075 | 108096 | 1295577 | 1447114 | 1472665 |

The optimal conditions were obtained by orthogonal analyses on the data of the experiment. Similarly, as shown in Table 3, when the concentration of MMPs and dilution ratios of antibody reached 300 µg/mL and 1/25, respectively, the fluorescence intensity was no longer increasing significantly. Thus, 300 µg/mL of MMPs and a dilution ratio of 1/25 was selected as the optimal condition for FT4 assay.
